# Prognostic significance of serum beta-2 microglobulin in patients with diffuse large B-cell lymphoma in the rituximab era

**DOI:** 10.18632/oncotarget.12734

**Published:** 2016-10-18

**Authors:** Seyoung Seo, Jung Yong Hong, Shinkyo Yoon, Changhoon Yoo, Ji Hyun Park, Jung Bok Lee, Chan-sik Park, Jooryung Huh, Yoonse Lee, Kyung Won Kim, Jin-Sook Ryu, Seok Jin Kim, Won Seog Kim, Dok Hyun Yoon, Cheolwon Suh

**Affiliations:** ^1^ Department of Oncology, Asan Medical Center, University of Ulsan College of Medicine, Seoul, Korea; ^2^ Department of Medical Statistics, Asan Medical Center, University of Ulsan College of Medicine, Seoul, Korea; ^3^ Department of Pathology, Asan Medical Center, University of Ulsan College of Medicine, Seoul, Korea; ^4^ Department of Otorhinolaryngology, Asan Medical Center, University of Ulsan College of Medicine, Seoul, Korea; ^5^ Department of Radiology, Asan Medical Center, University of Ulsan College of Medicine, Seoul, Korea; ^6^ Department of Nuclear Medicine, Asan Medical Center, University of Ulsan College of Medicine, Seoul, Korea; ^7^ Department of Hemato-oncology, Bundang Jesaeng Hospital, Gyeonggi-do, Korea; ^8^ Division of Hematology-Oncology, Department of Medicine, Samsung Medical Center, Sungkyunkwan University School of Medicine, Seoul, Korea

**Keywords:** beta 2-microglobulin, diffuse large B-cell lymphoma, rituximab, prognosis, non-Hodgkin lymphoma

## Abstract

The prognostic value of serum beta-2 microglobulin for diffuse large B-cell lymphoma (DLBCL) is not well known in the rituximab era. A retrospective registry data analysis of 833 patients with *de novo* DLBCL treated with rituximab, cyclophosphamide, doxorubicin, vincristine, and prednisone (R-CHOP) was conducted to establish the prognostic significance of serum beta-2 microglobulin at a ≥2.5 mg/L cutoff. Five-year progression-free survival (PFS, 76.1% vs. 41.0%; *p* < 0.001) and overall survival (OS, 83.8% vs. 49.2%; *p* < 0.001) were significantly worse in patients with elevated serum beta-2 microglobulin (*n* = 290, 34.8%). Furthermore, the five parameters of the International Prognostic Index, accompanying B symptoms, bone marrow involvement and impaired renal function were associated with worse PFS and OS. In multivariate analysis, elevated beta-2 microglobulin was a significant poor prognostic factor for PFS (hazard ratio [HR], 1.70; 95% confidence interval [CI], 1.29–2.24; *p* < 0.001) and OS (HR, 2.0; 95% CI, 1.47–2.75; *p* < 0.001). In an independent validation cohort of 258 R-CHOP treated patients with *de novo* DLBCL, elevated beta-2 microglobulin levels remained a significant poor prognostic factor for PFS (HR, 2.03; 95% CI, 1.23–3.32; *p* = 0.005) and exhibited a strong trend of association with worse OS (HR, 1.64; 95% CI, 0.98–2.75; *p* = 0.062). The significance of serum beta-2 microglobulin levels as an independent prognostic factor for patients with DLBCL receiving R-CHOP is confirmed.

## INTRODUCTION

Diffuse large B-cell lymphoma (DLBCL) is the most common subtype of non-Hodgkin lymphoma, a heterogeneous disease with a variety of molecular aberrations and diverse clinical outcomes [1, 2]. In recent years, significant advances in the treatment of DLBCL have been achieved with the addition of the anti-CD20 monoclonal antibody, rituximab to the existing protocol consisting of cyclophosphamide, doxorubicin, vincristine, and prednisone (CHOP) [3, 4].

The International Prognostic Index (IPI) utilized for over the past 20 years to determine the prognosis of patients with DLBCL, remains a valid predictor of clinical outcomes even in the rituximab-CHOP (R-CHOP) era [5, 6]. However, the standard IPI has suboptimal predictive power in high-risk patients with DLBCL and new prognostic tools such as the revised-IPI (R-IPI) and National Comprehensive Cancer Network (NCCN)-IPI showed to improve risk stratification of patients [7, 8]. However, there is still an unmet need for the identification of newer and better prognostic parameters.

Beta-2 microglobulin is synthesized in all nucleated cells and forms the light chain subunit of the major histocompatibility complex class I antigen [9, 10]. Free soluble beta-2 microglobulin can be detected in blood, urine, and cerebrospinal fluid, following its release from the cell surface or cytoplasm [11]. Specifically, measurement of serum beta-2 microglobulin is essential for baseline work up of multiple myeloma and follicular lymphoma patients [12–14]. Previous studies showed elevated serum beta-2 microglobulin was an independent poor prognostic factor in patients with DLBCL treated with CHOP or CHOP-like regimens [15, 16]. However, the prognostic value of beta-2 microglobulin in patients with DLBCL treated with rituximab containing regimens has not yet been fully investigated.

The present study has investigated the prognostic value of baseline serum beta-2 microglobulin in patients with DLBCL treated with R-CHOP immunochemotherapy and externally validated its prognostic impact in an independent validation cohort.

## RESULTS

### Correlations between beta-2 microglobulin and clinical features

A total of 833 patients with DLBCL who met the inclusion criteria were included in the analysis. Baseline characteristics of patients are summarized in Table 1. Median age was 58 (range, 16–69) years and male to female ratio was 1.3:1.0. Median serum beta-2 microglobulin level was 2.1 (range, 0.7–29.6) mg/L. Serum beta-2 microglobulin level of 2.5 mg/L was used to classify patients into two groups. Accordingly, there were 290 (34.8%) patients in high beta-2 microglobulin group.

**Table 1:** Baseline characteristics of patients

Characteristics	*N* (%)	Serum B2M, *n*(%)		*P* value
		< 2.5 mg/L *n* = 543 (%)	≥ 2.5 mg/L *n* = 290 (%)	
Age, Years				< 0.001
≤ 60	488 (58.6)	369 (68.0)	119 (41.0)	
> 60	345 (41.4)	174 (32.0)	171 (59.0)	
Sex				0.015
Male	475 (57.0)	293 (54.0)	182 (62.8)	
Female	358 (43.0)	250 (46.0)	108 (37.2)	
ECOG PS				< 0.001
0–1	762 (91.5)	526 (96.9)	236 (81.4)	
2–4	91 (8.5)	17 (3.1)	54 (18.6)	
Serum LDH				< 0.001
Normal	440 (52.8)	371 (68.3)	69 (23.8)	
Elevated	393 (47.2)	172 (31.7)	221 (76.2)	
Estimated GFR (mL/min/1.73 m^2^)				< 0.001
≥ 60	774 (92.9)	538 (99.1)	236 (81.4)	
< 60	59 (7.1)	5 (0.9)	54 (18.6)	
Ann Arbor stage				< 0.001
I–II	398 (47.8)	337 (62.1)	61 (21.0)	
III–IV	435 (52.2)	206 (37.9)	229 (79.0)	
Number of extranodal sites				< 0.001
0–1	534 (64.1)	412 (75.9)	122 (42.1)	
≥ 2	299 (35.9)	131 (24.1)	168 (57.9)	
Bone marrow				< 0.001
No involvement	708 (85)	502 (92.4)	206 (71.0)	
Involvement	125 (15)	41 (7.6)	84 (29.0)	
B symptoms				< 0.001
No	648 (77.8)	477 (87.8)	171 (59.0)	
Yes	185 (22.2)	66 (12.2)	119 (41.0)	
Hans classification^a^ (n = 693)				0.008
GCB	229 (33.0)	165 (36.5)	64 (26.6)	
Non-GCB	464 (67.0)	287 (63.5)	177 (73.4)	
Bulky disease				< 0.001
No	772 (92.7)	516 (95.0)	256 (88.3)	
Yes (> 10 cm)	61 (7.3)	27 (5.0)	34 (11.7)	
IPI				< 0.001
Low (0–1)	398 (47.8)	343 (63.2)	55 (19.0)	
Low-intermediate (2)	142 (17.0)	98 (18.0)	44 (15.2)	
High-intermediate (3)	149 (17.9)	62 (11.4)	87 (30.0)	
High (4–5)	144 (17.3)	40 (7.6)	104 (35.9)	
Revised IPI				< 0.001
Very good (0)	203 (24.4)	196 (36.1)	7 (2.4)	
Good (1–2)	337 (40.5)	245 (45.1)	92 (31.7)	
Poor (3–5)	293 (35.2)	102 (18.8)	191 (65.9)	
NCCN-IPI				< 0.001
Low (0–1)	138 (16.6)	132 (24.3)	6 (2.1)	
Low-intermediate (2–3)	394 (47.3)	300 (55.2)	94 (32.4)	
High-intermediate (4–5)	242 (29.1)	97 (17.9)	145 (50.0)	
High (≥6)	59 (7.1)	14 (2.6)	45 (15.5)	

Abbreviations: *B2M.* beta-2 microglobulin;*ECOG*, Eastern Cooperative Oncology Group;*GCB*, germinal center-like B cell-like; *GFR*, glomerular filtration rate; *IPI*, international prognostic index; *LDH*, lactate dehydrogenase;*NCCN*, National Comprehensive Cancer Network; *PS*, performance score; *UNL*, upper normal limit;

a Data for the Hans algorithm were available in 693 patients.

As shown in Table 1, the high beta-2 microglobulin group exhibited distinct adverse clinical features, such as older age (> 60), male sex, poor performance status (Eastern Cooperative Oncology Group [ECOG] performance score [PS] 2–4), elevated lactate dehydrogenase (LDH), impaired renal function estimated glomerular filtration rate [GFR] (< 60 mL/min/1.73 m^2^), advanced stage disease (stage III–IV), multiple extranodal involvement (≥ 2), presence of B-symptoms, non-germinal center B-cell-like (non-GCB) subtype, bone marrow involvement, bulky disease (> 10 cm), and higher IPI, R-IPI, and NCCN-IPI risk groups (Table 1).

### Prognostic significance of beta-2 microglobulin for survival

**Figure 1:**
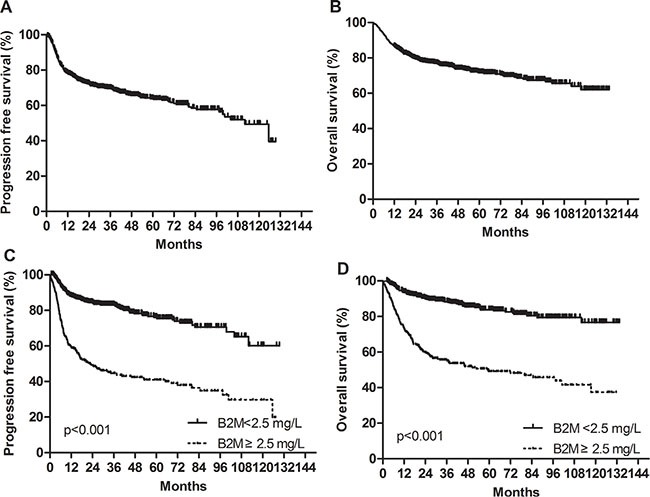
Progression-free survival and overall survival in the training cohort (**A**) Progression-free survival. (**B**) Overall survival. (**C**) Progression-free survival according to baseline serum beta-2 microglobulin levels. (**D**) Overall survival according to baseline serum beta-2 microglobulin levels.

With a median follow-up duration of 47.6 months (range, 12.0–133.5), median progression-free survival (PFS) and overall survival (OS) time points were not reached. The 5-year PFS and OS rates were 63.8% and 71.6%, respectively (Figure 1A and 1B). Patents with high serum beta-2 microglobulin had substantially worse 5-year PFS and OS than those with low beta-2 microglobulin (PFS, 41.0% vs. 76.1%; hazard ratio [HR], 3.59; 95% confidence interval [CI], 2.82–4.56; *p* < 0.001; OS, 49.2% vs. 83.8%; HR, 4.16; 95% CI, 3.16–5.48; *p* < 0.001, retrospectively) (Figure 1C and 1D).

**Figure 2:**
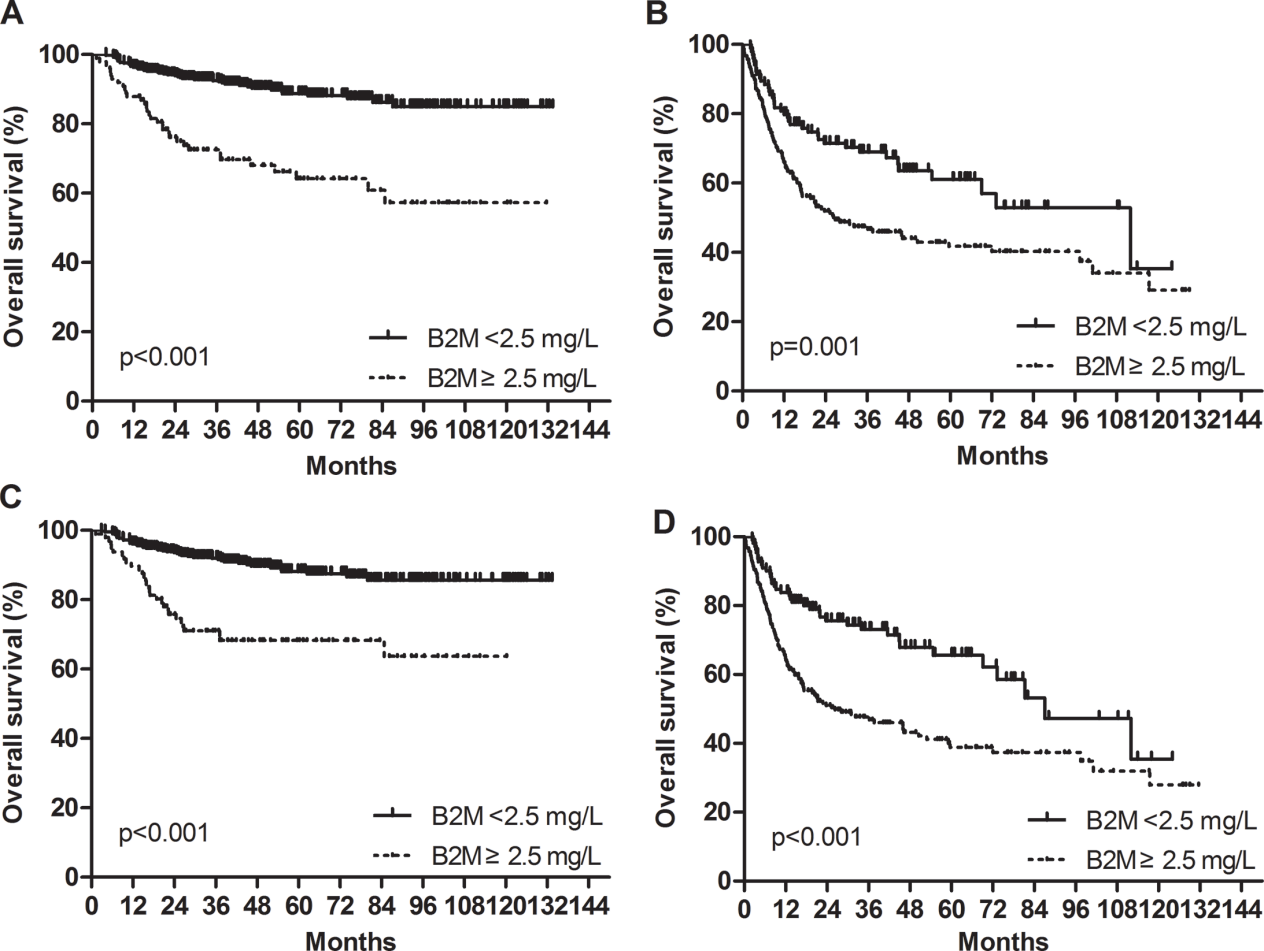
Impact of beta-2 microglobulin on the prediction of overall survival in the low/low-intermediate and high-intermediate/high risk groups by the IPI and NCCN-IPI in the training cohort (**A**) Low/low-intermediate risk groups by the IPI. (**B**) High-intermediate/high risk groups by the IPI. (**C**) Low/low-intermediate risk groups by the NCCN-IPI. (**D**) High-intermediate/high risk groups by the NCCN-IPI.

Further subgroup analysis was performed after according to the IPI and NCCN-IPI risk groups (low/low-intermediate [L/LI] vs. high-intermediate/high [HI/H]). Patients with high beta-2 microglobulin had significantly worse PFS and OS than those with low beta-2 microglobulin among both L/LI and HI/H subgroups. Specifically, subgroup analysis according to the IPI risk groups revealed that the 5-year OS rates of the low and high beta-2 microglobulin were 88.7% and 64.2% in the L/LI risk subgroups (*p* < 0.001) and 66.2% and 41.4% in the HI/H risk subgroups (*p* = 0.001), respectively (Figure 2A and 2B). Additional subgroup analysis based on NCCN-IPI risk groups determined that the 5-year OS rates of the low and high beta-2 microglobulin groups were 88.3% and 68.1% in the L/LI risk subgroups (*p* < 0.001) and 65.7% and 38.9% in the HI/H risk subgroups (*p* < 0.001), respectively (Figure 2C and 2D). When subgroup analysis based on accompanying renal impairment (estimated GFR < 60 mL/min/1.73 m^2^) was conducted, high serum beta-2 microglobulin retained its potent poor prognostic impact on 5-year PFS (42% vs. 75%; *p* < 0.001) and 5-year OS (50% vs. 84%; *p* < 0.001) in patients with normal renal function group. Among patents with impaired renal function, there was only a trend of worsening PFS and OS in patients with elevated serum beta-2 microglobulin without statistical significance (5- year PFS, 38.2% vs. 80.0%; *p* = 0.342 and 5-year OS, 44.0% vs. 100.0%; *p* < 0.055).

### Analysis of prognostic factors

Clinical factors associated with worse PFS and OS in the univariate analysis were as follows: older age (> 60 years), poor performance status (ECOG PS 2–4), elevated serum LDH, impaired renal function (estimated GFR < 60 mL/min/1.73 m^2^), advanced stage (stage III– IV), multiple extranodal involvement (≥ 2), presence of B-symptoms, bone marrow involvement, and non-GCB subtype (Table 2). Multivariate analysis showed that high beta-2 microglobulin group was associated significantly with worse PFS (HR, 1.70; 95% CI, 1.29–2.24; *p* < 0.001) and OS (HR, 2.00; 95% CI, 1.47–2.75; *p* < 0.001) (Table 3). Other independent prognostic factors for worse PFS and OS were older age (> 60 years), poor performance status (ECOG PS 2–4), elevated serum LDH, advanced stage (stage III–IV) (Table 3).

**Table 2:** Univariate analysis for the association between clinical factors and survival outcomes

Characteristics	Progression free survival		Overall survival	
	HR (95% CI)	*P* value	HR (95% CI)	*P* value
Serum B2M ≥ 2.5 mg/L	3.59 (2.82–4.56)	< 0.001	4.16 (3.16–5.48)	< 0.001
Age > 60 years	2.72 (2.13–3.46)	< 0.001	3.01 (2.28–3.95)	< 0.001
Female	0.89 (0.70–1.13)	0.327	0.99 (0.76–1.30)	0.962
ECOG PS, 2–4	3.99 (2.95–5.41)	< 0.001	4.17 (2.99–5.82)	< 0.001
Serum LDH > UNL	3.69 (2.83–4.80)	< 0.001	3.78 (2.80–5.10)	< 0.001
Ann Arbor stage, III–IV	3.20 (2.44–4.20)	< 0.001	3.49 (2.57–4.76)	< 0.001
Extranodal sites ≥ 2	2.72 (2.15–3.46)	< 0.001	2.93 (2.24–3.82)	< 0.001
B symptoms	2.37 (1.85–3.03)	< 0.001	2.39 (1.82–3.15)	< 0.001
Non-GCB type by the Hans algorithm^a^	1.40 (1.04–1.88)	0.026	1.50 (1.07–2.10)	0.019
Bone marrow involvement	1.91 (1.44–2.54)	< 0.001	1.82 (1.32–2.51)	< 0.001
Bulky disease > 10 cm	1.61 (1.10–2.35)	0.014	1.51 (0.98–2.33)	0.061
Estimated GFR < 60	2.26 (1.59-3.23)	< 0.001	2.52 (1.71-3.70)	< 0.001
IPI				
Low (0–1)	1		1	
Low-intermediate (2)	1.90 (1.29–2.79)	0.001	1.81 (1.14–2.87)	0.012
High-intermediate (3)	3.13 (2.22–4.42)	< 0.001	3.81 (2.59–5.61)	< 0.001
High (4–5)	7.29 (5.35–9.94)	< 0.001	7.88 (5.53–11.22)	< 0.001
Revised IPI				
Very good (0)	1		1	
Good (1–2)	3.44 (2.07–5.70)	< 0.001	2.91 (1.65–2.52)	< 0.001
Poor (3–5)	9.64 (5.93–15.69)	< 0.001	10.05(5.80–17.41)	< 0.001
NCCN-IPI				
Low (0–1)	1		1	
Low-intermediate (2–3)	2.48 (1.43–4.29)	0.001	2.98 (1.49–5.99)	0.002
High-intermediate (4–5)	7.12 (4.16–12.17)	< 0.001	9.10 (4.60–17.99)	< 0.001
High (≥ 6)	17.17 (9.60–30.72)	< 0.001	24.47 (11.90–50.32)	< 0.001

Abbreviations: *B2M*, beta-2 microglobulin; *CI*, confidence interval; *ECOG*, Eastern Cooperative Oncology Group; GCB, germinal center-like B cell-like; *HR*, hazard ratio; *IPI*, International Prognostic index *LDH*, lactate dehydrogenase; *NCCN*, National Comprehensive Cancer Network; *PS*, performance score; *UNL*, upper normal limit to.

a Data for the Hans algorithm were available in 693 patients.

**Table 3:** Mutivariate analysis for the prognostic impact of clinical factors on progression free survival and overall survival

	Progression-free survival		Overall survival	
	HR (95% CI)	*P* value	HR (95% CI)	*P* value
B2M (≥ 2.5 mg/L)	1.70 (1.29–2.24)	< 0.001	2.00 (1.47–2.75)	< 0.001
Age (> 60 years)	1.94 (1.50–2.50)	< 0.001	2.13 (1.60–2.83)	< 0.001
ECOG PS (3–4)	1.65 (1.19–2.28)	0.003	1.70 (1.18–2.39)	0.003
LDH (> UNL)	2.12 (1.57–2.87)	< 0.001	1.96 (1.39–2.77)	< 0.001
Ann Arbor stage ≥ 3	1.42 (1.19–2.20)	0.002	1.68 (1.18–2.39)	0.004

Abbreviations: *B2M*, beta-2 microglobulin;*CI*, confidence interval; *ECOG*, Eastern Cooperative Oncology Group; *HR*, hazard ratio; *LDH*, lactate dehydrogenase; *PS*, performance score; *UNL*, upper normal limit.

### External validation in validation cohort

Between August 2010 and August 2012, 595 patients with DLBCL were enrolled in a prospective multicenter cohort study, the Prospective Cohort Study with Central Nervous System Evaluation in DLBCL (PROCESS) study (ClinicalTrials.gov identifier: NCT01202448) which included 26 institutes participating in the Consortium for Improving Survival of Lymphoma (CISL) in Korea [17]. All patients were treated with at least one cycle of R-CHOP. Eighty-two patients enrolled from our institution were excluded for avoiding data duplication. Baseline serum beta-2 microglobulin levels were measured in 258 (50.3%) patients from 12 institutions by radioimmunoassay and these 258 patients were included in the external validation cohort in the current study.

The median value of beta-2 microglobulin in the validation cohort consisting of 258 patients with DLBCL was 2.1 (range, 0.5–25.7) mg/L. In 84 patients (32.6%), the baseline beta-2 microglobulin levels were founded to be elevated. Patients in the training cohort were slightly younger than those in the validation cohort (median age, 58 vs. 61 years) and had better ECOG PS (PS 0–1, 91.5% vs. 86.8%) and less frequent B-symptoms (22.2% vs. 28.7%). Other baseline characteristics were not significantly different between the training cohort and the validation cohort (Supplementary Table S1). With a median follow-up duration of 34.7 months (range, 16.0–52.2), median PFS and OS values could not be reached. The 3-year PFS and OS rates were 71.8% and 69.3%, respectively in the validation cohort (Supplementary Figure S1A and S1B). Univariate analysis showed that patients with elevated beta-2 microglobulin levels had significantly worse PFS (HR, 3.34; 95% CI, 2.15–5.21; *p* < 0.001) and OS (HR, 3.01; 95% CI, 1.99–4.78; *p* < 0.001) (Supplementary Table S2, Supplementary Figure S1C and S1D). Furthermore, multivariate analysis including confounding variables such as older age (> 60 years), poor performance status (ECOG PS 2–4), elevated serum LDH, advanced disease stage (stage III–IV), multiple extranodal involvement (≥ 2), presence of B-symptoms, and bone marrow involvement showed that high beta-2 microglobulin retained its significant poor prognostic impact for PFS (HR, 1.93; 95% CI, 1.18–3.18; *p* = 0.009) and exhibited a strong trend toward worse OS with borderline statistical significance (HR, 1.64; 95% CI, 0.98–2.75; *p* = 0.062) (Table 4).

**Table 4:** Clinical factors prognostic of progression free survival and overall survival by multivariate selection in the validation cohort

	Progression-free survival		Overall survival	
	HR (95% CI)	*P* value	HR (95% CI)	*P* value
B2M (≥ 2.5 mg/L)	1.93 (1.18–3.18)	0.009	1.64 (0.98–2.75)	0.062
Age (> 60 years)	2.06 (1.28–3.32)	0.003	2.47 (1.48–4.11)	0.001
ECOG PS (3–4)	3.00 (1.80–5.01)	< 0.001	2.90 (1.68–4.99)	< 0.001
LDH (> UNL)	1.68 (1.00–2.83)	0.049	1.88 (1.09–3.24)	0.023

Abbreviations: *B2M*, beta-2 microglobulin;*CI*, confidence interval; *ECOG*, Eastern Cooperative Oncology Group; *HR*, hazard ratio; *LDH*, lactate dehydrogenase; *PS*, performance score; *UNL*, upper normal limit.

## DISCUSSION

In this retrospective cohort study, the elevated baseline serum beta-2 microglobulin was associated with distinct adverse clinical features and higher IPI, R-IPI and NCCN-IPI risk groups in patients with DLBCL treated with R-CHOP. Moreover, the elevated baseline serum beta-2 microglobulin was found to be a potent independent poor prognostic factor for patients with DLBCL in the rituximab era.

Recent advances in biology, immunology, and genomics identified novel biomarkers of lymphoma such as oncogenic proteins, biologic pathways, and genetic mutations [2, 18–21]. However, high costs, long turnaround time, and methodological complexities remain big hurdles for widespread adoption of biological technologies in real-life clinical practice.

Serum beta-2 microglobulin is a simple, inexpensive, and standardized measurable parameter. As well-established prognostic factor, high serum beta-2 microglobulin is a component of the International Staging System (ISS) and Revised-ISS for multiple myeloma [12, 13, 22, 23] and one of the parameters of the follicular lymphoma international prognostic index 2, a proven prognostic model of follicular lymphoma in the rituximab era [14]. Previous studies suggested serum beta-2 microglobulin level as a potential prognostic biomarker of diverse lymphoproliferative disorders, including Hodgkin lymphoma [24, 25], mucosa-associated lymphoid tissue lymphoma [26, 27], DLBCL prior to the rituximab era [16, 28], mantle cell lymphoma [29, 30], and nasal NK/T-cell lymphoma [31, 32]. The biological mechanisms underlying the association between elevated serum beta-2 microglobulin and poor prognosis are not completely understood. Finding from previous studies suggested that serum beta-2 microglobulin might indicate cell turnover rate, high tumor burden and subsequent unfavorable clinical course of cancer [11, 33].

The prognostic role of beta-2 microglobulin in patients with DLBCL treated with rituximab has not been fully investigated in previous studies except for the notable Japanese studies by Kanemsa et al. and Miyashita et al. [34, 35]. They reported that elevated serum beta-2 microglobulin was a poor prognostic predictor in patients with DLBCL in the rituximab era. However, there are several limitations that should be considered in the interpretation of these two studies. First, there was a discrepancy in the cutoff values of serum beta-2 microglobulin used between the two studies. Both groups used receiver operating characteristic curve (ROC) analysis for predicting binary outcome (death or alive) and PFS, respectively with 3.2 mg/L and 1.75 mg/L as cutoff values. As time-dependent ROC analysis is considered as more reliable than ROC analysis with binary outcomes in assessing parameters as potential prognostic factors, the present study focused on the cutoff value reported by Miyashita *et al.* However, they reported that this cutoff had < 50% specificity, although the sensitivity was > 80%. Second, neither study considered renal impairment as a potential confounder. Given that beta-2 microglobulin is excreted mainly via kidneys, renal failure itself might lead to increased serum beta-2 microglobulin levels. Finally, neither study included further validation on external cohorts.

The current study aimed to identify a more reliable cutoff value using a statistical technique outlined by Contal *et al.* and found that the cutoff serum beta-2 microglobulin level of ≥ 2.4 mg/L was the best predictor of OS [36]. This value was very close to the manufacturer's upper normal limit of ≥ 2.5 mg/L; thus, serum beta-2 microglobulin value of ≥ 2.5 mg/L was used as the cutoff value for easy adoption of beta-2 microglobulin levels in real-life clinical practice. In current study, the subgroup analysis was performed according to renal function status. In the subgroup with normal renal function, elevated serum beta-2 microglobulin retained a significant prognostic predictor for worse PFS and OS. In the subgroup with impaired renal function, almost all patients had elevated serum beta-2 microglobulin levels and only five patients had normal beta-2 microglobulin levels. In this subgroup, although, a strong trend toward worse PFS and OS rates existed among patients with elevated serum beta-2 microglobulin levels, statistically significance could not reached due to the small number of patients with normal beta-2 microglobulin levels. Thus, the findings of the current study suggest that elevated serum beta-2 miroglobulin might reflect poor prognosis even in patients with DLBCL with impaired renal function. A subset of patients with DLBCL were enrolled in the PROCESS study, used as external validation cohort in current study, confirmed that elevated beta-2 microglobulin level (≥ 2.5 mg/L) was a predictor of worse PFS and OS rates.

To the best of our knowledge, this is the largest study to demonstrate the prognostic significance of serum beta-2 microglobulin in patients with DLBCL treated with rituximab and the first study to validate its prognostic impact in an independent validation cohort. Moreover, serum beta-2 microglobulin level performed well as a prognostic predictor in risk groups (L/LI vs. HI/H) of both IPI and NCCN-IPI, suggesting that beta-2 microglobulin might be a better prognostic indicator than other elements of the IPI, especially external nodal involvement which had no statistical significance in multivariate analysis in current study.

However, there are several limitations of present study that need to be addressed. First, this was retrospective study, even though prospective cohorts were used (Asan Medical Center registry as the training cohort and PROCESS study as the validation cohort). Second, the sample size of the validation cohort was small due to missing data including beta-2 microglobulin values. Finally, the follow-up duration of validation cohort was relatively shorter than that of the training cohort.

In summary, this study demonstrated that patients with DLBCL with elevated serum beta-2 microglobulin showed distinct adverse clinical features and followed a significantly worse clinical course. Further analysis confirmed the significance of serum beta-2 microglobulin as an independent prognostic factor for patient with DLBCL receiving R-CHOP immunochemotherapy. Further studies are needed to determine whether a modified prognostic index that incorporate serum beta-2 microglobulin will show improved performance in patients with DLBCL.

## MATERIALS AND METHODS

### Patients

We identified 940 patients diagnosed with DLBCL in Asan Medical Center lymphoma registry between January 2004 and April 2014. A total of 833 patients who met the following criteria were included in this study: (1) pathologically confirmed DLBCL according to the World Health Organization (WHO) classification, (2) administration of R-CHOP as first-line treatment, (3) available serum beta-2 microglobulin measurement at diagnosis. Patients with unknown baseline serum beta-2 microglobulin levels, patients with primary central nervous system lymphoma, and those who were positive for human immunodeficiency virus were excluded from the study. Clinical data including disease and survival status were updated in June 2015. This study was approved by the institutional review boards at Asan Medical Center, in accordance with the Declaration of Helsinki.

### Clinical characteristics and treatment

Clinical characteristics obtained were age, sex, ECOG PS, LDH, serum creatinine, Ann Arbor stage, extranodal involvement, bone marrow involvement, presence of B-symptoms, and presence of bulky disease (> 10 cm). The IPI, R-IPI, and NCCN-IPI risk groups were estimated using pretreatment variables including age, LDH, ECOG PS, Ann Arbor stage, and extranodal involvement [5, 7, 8].

First-line treatment consisted of standard R-CHOP therapy (375 mg/m^2^ rituximab, 750 mg/m^2^ cyclophosphamide, 50 mg/m^2^ doxorubicin, and 1.4 mg/m^2^ [maximum 2.0 mg/body] vincristine on day 1, and 100 mg prednisolone on days 1–5 for 21 days per cycle). Treatment response was assessed using the revised response criteria [37].

### Pathology

The pathology of the patients with DLBCL was confirmed by an expert hematopathologist (J.H.) using the WHO classification. For immunohistochemical staining, a panel of monoclonal antibodies against CD20, CD3, CD10, BCL-6, BCL-2, IRF4/MUM-1, and Ki67 were used. Patients were classified into GCB and non-GCB subtypes using the Hans algorithm [38].

### Determination of serum beta-2 microglobulin

Serum beta-2 microglobulin levels were determined using a radioimmunoassay kit (Immunotech, Prague, Czech Republic), according to the manufacturer's instructions. The optimal cutoff point of serum beta-2 microglobulin level was estimated at 2.4 mg/L using the methods by Contal *et al.*, that maximizes the difference between two groups by a defined cutoff value [36] (Supplementary Table S3). This value was very close to the manufacturer's upper normal limit (≥ 2.5 mg/L), and the prognostic impact of beta-2 microglobulin according to the cutoff values of 2.4 and 2.5 mg/L were comparable by multivariate analysis (Supplementary Table S4). Thus, the serum beta-2 microglobulin level of ≥ 2.5 mg/L was used as the cutoff value to dichotomize patients into low beta-2 microglobulin (< 2.5 mg/L) and high beta-2 microglobulin (≥ 2.5 mg/L) groups for further analysis.

### Statistical analysis

Categorical variables were evaluated using the chi-square test or Fisher's exact test, as appropriate. PFS was defined as the time from diagnosis to tumor recurrence, progression or death from any cause, and OS was calculated from the date of diagnosis to death. PFS and OS were censored on the last date of follow-up. The Kaplan–Meier analysis was used to estimate PFS and OS, and survival curves were compared by the log-rank test. The Cox proportional hazards model was used to estimate HRs for survival outcomes. *P* values of <0.05 were considered statistically significant, and two-sided tests were used in all analyses. Statistical analyses were performed using the Statistical Package for the Social Sciences version 21 (IBM Co., Armonk, NY).

## SUPPLEMENTARY MATERIALS FIGURES AND TABLES


