# Supplementary material for: Prognostic significance of serum beta-2 microglobulin in patients with diffuse large B-cell lymphoma in the rituximab era
Source: Oncotarget. 2016 Oct 18;7(47):76934–43. doi: 10.18632/oncotarget.12734 (PMC5363560; doi:10.18632/oncotarget.12734)
Supplement: Supplementary file 1 [file oncotarget-07-76934-s001.pdf]

Prognostic significance of serum beta-2 microglobulin in patients with diffuse large B-cell lymphoma in the rituximab era

Supplementary Materials

Supplementary Table S1: Baseline characteristics in the training set and validation set

	Training set *n* = 833 (% of cohort)	Validation set *n* = 258 (% of cohort)	*P* value
Age, Years			0.008
≤ 60	488 (58.6)	127 (49.2)	
> 60	345 (41.4)	131 (50.8)	
Sex			0.751
Male	475 (57.0)	150 (58.1)	
Female	358 (43.0)	108 (41.9)	
ECOG PS			0.027
0–1	762 (91.5)	224 (86.8)	
2–4	91 (8.5)	34 (13.2)	
Serum LDH			0.148
Normal	440 (52.8)	123 (47.7)	
Elevated	393 (47.2)	135 (52.3)	
Serum B2M			0.505
< 2.5 mg/L	543 (65.2)	174 (67.4)	
≥ 2.5 mg/L	290 (34.8)	84 (32.6)	
Ann Arbor stage			0.766
I–II	398 (47.8)	126 (48.8)	
III–IV	435 (52.2)	132 (51.2)	
Extranodal sites			0.523
0–1	534 (64.1)	171 (66.3)	
≥ 2	299 (35.9)	87 (33.7)	
Bone marrow			0.568
No involvement	708 (85)	223 (86.4)	
Involvement	125 (15)	35 (13.6)	
B symptoms			0.033
No	648 (77.8)	184 (71.3)	
Yes	185 (22.2)	74 (28.7)	
IPI			0.185
Low (0–1)	398 (47.8)	106 (41.1)	
Low-intermediate (2)	142 (17.0)	57 (22.1)	
High-intermediate (3)	149 (17.9)	49 (19.0)	
High (4–5)	144 (17.3)	46 (17.8)	
Revised IPI			0.013
Very good (0)	203 (24.4)	41 (15.9)	
Good (1–2)	337 (40.5)	122 (47.3)	
Poor (3–5)	293 (35.2)	95 (36.8)	

Abbreviations: *B2M*, beta-2 microglobulin; *ECOG*, Eastern Cooperative Oncology Group; *IPI*, International Prognostic index; *LDH*, lactate dehydrogenase; *PS*, performance score; *UNL*, upper normal limit.

Supplementary Table S2: Univariate analysis of the association between clinical factors and survival outcomes in the validation set

Characteristics	Progression free survival		Overall survival	
	**HR (95% CI)**	***P* value**	**HR (95% CI)**	***P* value**
Serum B2M ≥ 2.5 mg/L	3.34 (2.15–5.21)	< 0.001	4.16 (3.16–5.48)	< 0.001
Age > 60 years	2.37 (1.48–3.78)	< 0.001	2.73 (1.66–4.51)	< 0.001
Female	0.81 (0.51–1.27)	0.349	0.82 (0.51–1.31)	0.403
ECOG PS, 2–4	4.35 (2.69–7.05)	< 0.001	4.09 (2.45–6.81)	< 0.001
Serum LDH > UNL	2.51 (1.55–4.05)	< 0.001	2.61 (1.57–4.34)	< 0.001
Ann Arbor stage, III–IV	2.02 (1.27–3.19)	0.003	2.02 (1.24–3.27)	0.005
Extranodal sites ≥ 2	2.06 (1.55–4.05)	< 0.001	1.93 (1.22–3.06)	0.005
B symptoms	1.51 (0.96–2.40)	0.078	1.65 (1.02–2.65)	0.040
Bone marrow involvement	2.49 (1.48–4.16)	0.001	2.47 (1.45–4.21)	0.001
IPI				
Low	1		1	
Low-intermediate	1.93 (0.98–3.82)	0.059	1.63 (0.78–3.38)	0.193
High-intermediate	3.03 (1.59–5.79)	0.001	2.74 (1.40–5.37)	0.003
High	5.26 (2.86–9.68)	< 0.001	5.43 (2.91–10.14)	< 0.001
Revised IPI				
Very good	1		1	
Good	5.79 (1.39–24.20)	0.016	4.96 (1.18–20.87)	0.029
Poor	13.78 (3.34–56.76)	< 0.001	12.48 (3.02–51.50)	< 0.001

Abbreviations: *B2M,* beta-2 microglobulin; *ECOG*, Eastern Cooperative Oncology Group; *HR*, hazard ratio; *IPI*, International Prognostic index; *LDH*, lactate dehydrogenase; *PS*, performance score; *UNL*, upper normal limit.

Supplementary Table S3: Cox proportional hazard regression model results for various cutoff points of serum beta-2 microglobulin level to predict overall survival

Beta-2 microglobulin (mg/L)	Hazard ratio	*P*-value	Omnibus test of model coefficient: Chi-square
2	4.344	< 0.001	82.475
2.1	4.648	< 0.001	100.532
2.2	4.377	< 0.001	107.109
2.3	4.408	< 0.001	107.109
2.4	4.430	< 0.001	123.355
2.5	4.163	< 0.001	121.990
2.6	3.574	< 0.001	99.426
2.7	3.661	< 0.001	105.315
2.8	3.602	< 0.001	103.021
2.9	3.461	< 0.001	95.816
3	3.414	< 0.001	92.434
3.1	3.394	< 0.001	90.586
3.2	3.438	< 0.001	90.904
3.3	3.489	< 0.001	90.844
3.4	3.588	< 0.001	92.203
3.5	3.614	< 0.001	91.457
3.6	3.537	< 0.001	84.107
3.7	3.647	< 0.001	84.607
3.8	3.724	< 0.001	82.507
3.9	3.918	< 0.001	87.658
4.0	3.942	< 0.001	86.197

Supplementary Table S4: Clinical factors prognostic of progression free survival and overall survival from multivariate analysis in the training set with beta-2 microglobulin cutoff point of ≥ 2.4 mg/L

Characteristics	Progression free survival		Overall survival	
	HR (95% CI)	*P* value	HR (95% CI)	*P* value
B2M (≥ 2.4 mg/L)	1.97 (1.48–2.63))	< 0.001	2.16 (1.55–3.00)	< 0.001
Age (> 60 years)	1.88 (1.46–2.43)	< 0.001	2.09 (1.57–2.77)	< 0.001
ECOG PS (3–4)	1.64 (1.19–2.26)	0.003	1.70 (1.20–2.42)	0.003
LDH (> UNL)	2.01 (1.49–2.72)	< 0.001	1.89 (1.34–2.67)	< 0.001
Ann arbor Stage ≥ 3	1.58 (1.16–2.45)	0.004	1.68 (1.18–2.38)	0.004

Abbreviations: *B2M*, beta-2 microglobulin; *ECOG,* Eastern Cooperative Oncology Group; *LDH,* lactate dehydrogenase; *PS*, performance score; *UNL*, upper normal limit.

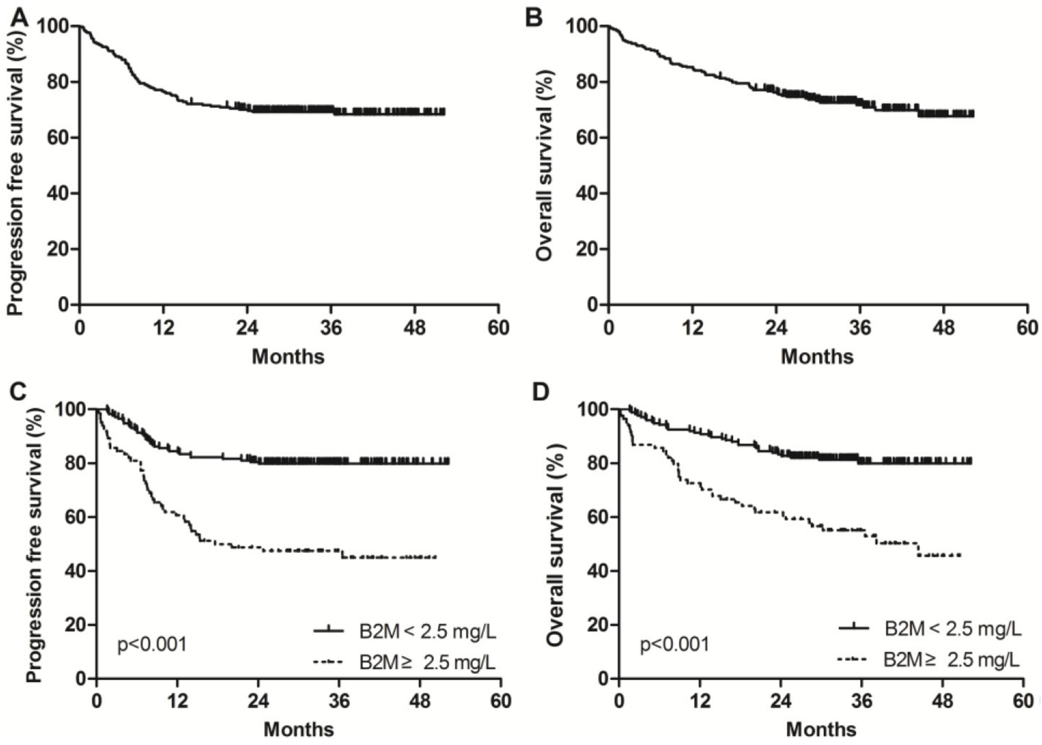

Supplementary Figure S1: Progression free and overall survival in the validation cohort. (**A**) Progression free survival. (**B**) Overall survival. (**C**) Progression free survival according to baseline serum beta-2 microglobulin levels. (**D**) Overall survival according to baseline serum beta-2 microglobulin levels.
